# Inhibition of p38 MAPK Mitigates Lung Ischemia Reperfusion Injury by Reducing Blood–Air Barrier Hyperpermeability

**DOI:** 10.3389/fphar.2020.569251

**Published:** 2020-12-11

**Authors:** Tiantian Wang, Chunxia Liu, Ling-hui Pan, Zhen Liu, Chang-long Li, Jin-yuan Lin, Yi He, Jing-yuan Xiao, Siyi Wu, Yi Qin, Zhao Li, Fei Lin

**Affiliations:** ^1^ Department of Anesthesiology, Guangxi Medical University Cancer Hospital, Nanning, China; ^2^ Department of Experimental Research, Guangxi Medical University Cancer Hospital, Nanning, China

**Keywords:** p38 mitogen-activated protein kinase, lung ischemia reperfusion injury, blood-air barrier hyperpermeability, zonulae occludente 1, vascular endothelial cadherin

## Abstract

**Background:** Lung ischemia reperfusion injury (LIRI) is a complex pathophysiological process activated by lung transplantation and acute lung injury. The p38 mitogen-activated protein kinase (MAPK) is involved in breakdown of the endothelial barrier during LIRI, but the mechanism is still unclear. Therefore, we investigated the function of p38 MAPK in LIRI *in vivo* and *in vitro*.

**Methods:** Sprague–Dawley rats were subjected to ischemia reperfusion with or without pretreatment with a p38 MAPK inhibitor. Lung injury was assessed using hematoxylin and eosin staining, and pulmonary blood–air barrier permeability was evaluated using Evans blue staining. A rat pulmonary microvascular endothelial cell line was infected with lentiviral expressing short hairpin (sh)RNA targeting p38 MAPK and then cells were subjected to oxygen/glucose deprivation and reoxygenation (OGD/R). Markers of endothelial destruction were measured by western blot and immunofluorescence.

**Results:** *In vivo* LIRI models showed structural changes indicative of lung injury and hyperpermeability of the blood–air barrier. Inhibiting p38 MAPK mitigated these effects. Oxygen/glucose deprivation and reoxygenation promoted hyperpermeability of the endothelial barrier *in vitro*, but knockdown of p38 MAPK attenuated cell injury; maintained endothelial barrier integrity; and partially reversed injury-induced downregulation of permeability protein AQP1, endothelial protective protein eNOS, and junction proteins ZO-1 and VE-cadherin while downregulating ICAM-1, a protein involved in destroying the endothelial barrier, and ET-1, a protein involved in endothelial dysfunction.

**Conclusion:** Inhibition of p38 MAPK alleviates LIRI by decreasing blood–air hyperpermeability. Blocking p38 MAPK may be an effective treatment against acute lung injury.

## Introduction

Lung ischemia reperfusion injury (LIRI) is a complex pathophysiological process (Lau et al., 2009). LIRI is characterized by increased pulmonary vascular resistance, increased microvascular permeability, pulmonary edema, pulmonary hypertension, and impaired oxygenation (de Perrot et al., 2003). Many clinical conditions, including lung transplantation, acute lung injury, and acute respiratory distress syndrome, can induce LIRI, which is a risk factor for late graft failure in lung transplantation (Lee et al., 2010; Sun et al., 2017).

LIRI disrupts the blood–air barrier at the interface of an alveolar epithelial cell layer and endothelial cell layer, promoting lung inflammation and acute lung injury (Zhang et al., 2015; Bärnthaler et al., 2017; Kling et al., 2017). Current methods to prevent breakdown of the blood–air barrier are ineffective; thus, it is crucial to clarify the mechanisms of blood–air barrier dysfunction in LIRI.

Microvascular endothelial cells are targeted for blood–air barrier destruction during LIRI (Zhang et al., 2015), and several proteins may be dysregulated during disruption of the blood–air barrier. AQP1, a water-selective channel protein, is upregulated when lung endothelial cells are more permeable to water (Ma et al., 2004); mice lacking AQP1 show reduced movement of endothelial fluid, and their microvascular endothelium is less permeable to water (Towne et al., 2000; Hua et al., 2019). eNOS/NO signaling, an endothelial homeostasis protective pathway, improved endothelial barrier during ischemia/reperfusion through suppressing inflammation (Qi et al., 2016; Zhang et al., 2019). Conversely, overexpression of ET-1, an endothelial dysfunction maker protein, promoted endothelial permeability during short-term ischemia with long-term reperfusion (Zhang et al., 2013). ICAM-1 plays an important role in destroying the endothelial barrier (Wang et al., 1999). It increases permeability of human dermal microvascular endothelial cells by changing their cell junctions or cytoskeleton organization (Clark et al., 2007). Endothelial cells form tight barriers through tight junctions in a process regulated by the protein ZO-1 (Tornavaca et al., 2015), and VE-cadherin is an adhesion protein specifically located at tight junctions (Li et al., 2019). F-actin is the main cytoskeletal protein involved in cellular contraction, which is important for maintaining normal morphology and function (Deng et al., 2018). LIRI induces degradation of tight junctions by downregulating ZO-1 and VE-cadherin while stress fibers accumulate, compromising the endothelial barrier (Kim et al., 2020).

Inflammation during LIRI has been linked to activation of p38 mitogen-activated protein kinase (MAPK), which can influence cell differentiation, proliferation, and survival and production of proinflammatory mediators (Boyle et al., 2006; Wada et al., 2017; Xiao et al., 2017). Although p38 MAPK is known to help damage the endothelial barrier during inflammation-induced acute lung injury (Tan et al., 2013; Li et al., 2015; Song et al., 2017), whether and how it contributes to blood–air barrier hyperpermeability is unclear.

We hypothesized that p38 MAPK participates in the process of LIRI by altering expression of proteins that regulate permeability of the blood–air barrier. Specifically, we speculated that inhibiting p38 MAPK may upregulate AQP1 while downregulating ICAM-1, thereby reversing LIRI-induced changes in expression of ZO-1, VE-cadherin, and F-actin. We tested this possibility *in vivo* and *in vitro*.

## Materials and Methods

### Animals

Animal protocols were approved by the Institutional Animal Care and Use Committee of Guangxi Medical University (Nanning, China). Adult male Sprague–Dawley rats (220–250 g, 7–8 weeks) were acquired from the animal center of Guangxi Medical University. Animals were maintained in a 12 h light–dark cycle in humidity-controlled rooms with freely accessible food and water.

### Lung Ischemia Reperfusion Injury Model

Rats were randomly divided into four groups (*n* = 15 per group): DMSO, SB203580 (p38 MAPK inhibitor, dissolved in DMSO), I/R (ischemia reperfusion), and I/R + SB203580. The rats in the control and the SB203580 groups received DMSO as a vehicle control or SB203580 (10 mg/kg) by intraperitoneal injection 30 min before the sham surgery (Liu et al., 2008; Xiong et al., 2016; Zhang et al., 2020a). LIRI was induced in the I/R and I/R + SB203580 groups. Rats were sedated and mechanically ventilated using an ALC-V9A–type small animal ventilator (ALCOTT BIOTECH of Shanghai, China). Inspiratory-to-expiratory ratio was set to 1:1 and respiration rate to 60 breaths/min. The tidal volume was 8–10 ml/kg with the required fraction of inspired oxygen of 100%. In the I/R and I/R + SB203580 groups, rats were subjected to thoracotomy, and the left hilus pulmonis was occluded for 1 h using a vascular clamp. Then, the clamp was removed, and reperfusion was allowed for 4 h before sacrifice by bloodletting through the carotid artery (Fei et al., 2017). The rats in the I/R and I/R + SB203580 groups received DMSO as vehicle control or SB203580 by intraperitoneal injection 30 min before surgery.

### Hematoxylin-Eosin Staining

Lung tissue specimens were fixed in 4% paraformaldehyde and then embedded in paraffin. Samples were stained with hematoxylin and eosin and photographed under a light microscope (EVOS FL AutoLife Technologies). Lung injury was scored as previously described (Fei et al., 2017). Briefly, scoring was based on the infiltration of inflammatory cells, the extent of pulmonary edema, and the interstitial congestion and hemorrhage. For each mouse, six fields were assessed at 400X magnification. The score of lung injury is based on three criteria: aggregation or infiltration of inflammatory cells in vessel walls or air spaces [1 point = only wall, 2 = rare cells in air space, 3 = intermediate, and 4 = severe (air space congested)], hyaline membrane formation and interstitial congestion in the lung [1 point = normal lung, 2 = moderate (>25% of lung section), 3 = intermediate (25%–50% of lung section), and 4 = severe (>50% of lung section)], and presence (1) or absence (0) of hemorrhage. The scores for each criterion were summed to obtain the score for each animal. Six sections were independently assessed by two pathologists blinded to animal grouping.

### Transmission Electron Microscopy

Lung tissue samples were minced into pieces approximately 1 mm and fixed in 3% glutaraldehyde for at least 2 h, followed by 1% osmic acid for 1–2 h. Tissue was dehydrated in different concentrations of acetone before embedding in resin. Samples were cut into ultrathin sections with an ultramicrotome and observed with an H-7560 transmission electron microscope (H-7560, Tokyo, Japan).

### Lung Wet-To-Dry Ratio

After sacrifice, wet left lung tissue was weighed, incubated at 60°C for 96 h, and then weighed again. These values were used to calculate wet-to-dry ratios (Dai et al., 2015).

### Total Protein in Bronchoalveolar Lavage Fluid

After the right lung hilum was clipped, the left lung was flushed three times with 5 ml of cold phosphate-buffered saline (PBS) using a tracheal catheter to collect BALF (Fei et al., 2017). Total protein in the BALF was detected by a bicinchoninic acid (BCA) protein assay kit following the manufacturer’s instructions (Beyotime Biotechnology, China).

### Evans Blue Staining

Next, 0.5% Evans blue (4 mg/kg) was injected via the jugular vein 1 h before sacrifice. Then, the left lung tissue was weighed, homogenized, treated with formamide (100 mg/ml; Sigma-Aldrich, Shanghai, China), incubated at 37°C for 24 h, and centrifuged for 20 min at 10,000 *g*. Absorbance of the supernatant was measured at 620 nm, and the concentration of Evans blue was calculated from the absorbance according to a standard curve.

### Enzyme-Linked Immunosorbent Assay (ELISA)

After sacrifice, lung tissue was homogenized. The concentrations of IL-6 and IL-1β in lung tissues or in BALF (acquired as previously described in the total protein in bronchoalveolar lavage fluid section) were quantified using ELISA kits (Elabscience Biotechnology, Wuhan, China) following the manufacturer’s instructions.

### Immunohistochemistry

Lung tissue specimens were embedded in paraffin and sliced 2–4 μm thick. The sections were dewaxed, rehydrated, immersed in 0.01 M citrate buffer (pH 6.0), and boiled in a pressure cooker for 2 min. The sections were blocked with 3% hydrogen peroxide for 10 min and then incubated overnight at 4°C with rabbit monoclonal antibody against ZO-1 (1:400; catalog no. ab221547, Abcam, UK) or rabbit polyclonal antibody against VE-cadherin (1:100; catalog no. ab231227, Abcam). The sections were exposed to bio-goat antirabbit IgG as a secondary antibody from an SP (mouse/rabbit IgG)-POD kit (Catalog no. SP0041, Solarbio Life Sciences) following the manufacturer's instructions. Then, the sections were stained with DAB from the SP (mouse/rabbit IgG)-POD kit. Normal rabbit IgG (1:100, catalog no. AB-105-C, R&D) in PBS was used instead of a primary antibody (ZO-1 or VE-cadherin) in the immunohistochemistry procedure as a negative control. Spleen and kidney tissues instead of lung tissue were used for ZO-1 and VE-cadherin IHC as a positive control.

Expression of ZO-1 and VE-cadherin was visualized by a light microscope (EVOS FL AutoLife Technologies) and quantified from six randomly selected fields of view using Image-Pro-Plus 6. The integrated optical density (IOD) was measured from areas of protein staining, and the ratio of IOD area was used in statistical analysis.

### Knockdown of p38 Mitogen-Activated Protein Kinase *In Vitro*

Rat pulmonary microvascular endothelial cells (rPMVECs) were purchased from the BeNa Culture Collection (catalog no. BNCC-338210; Beijing, China) and cultured in DMEM (Gibco, China) supplemented with 10% fetal bovine serum (FBS; Gibco, Australia), 100 μg/ml penicillin/streptomycin (Gibco) and 1% endothelial cell growth supplement (ScienCell) at 37°C in an atmosphere of 5% CO_2_ and 95% air. rPMVECs were infected with lentivirus expressing short hairpin (sh)RNA against p38 MAPK (5’-GAC​CGT​TTC​AGT​CCA​TCA​T-3’) or a control shRNA (5’-TTC​TCC​GAA​CGT​GTC​ACG​T-3’) in the presence of polybrene (5 μg/ml). Cells were cultured for 72 h after infection. The lentiviruses were synthesized by Genechem (Shanghai, China).

### Cellular Oxygen/Glucose Deprivation and Reoxygenation

To mimic ischemic-like conditions *in vitro*, we subjected rPMVECs to OGD/R as described (Shen et al., 2004). Briefly, rPMVECs were washed three times with PBS, fed serum- and glucose-free medium (Gibco), and then placed for 1 h at 37°C in a Whitley H35 Hypoxystation (Don Whitley Scientific) in an atmosphere of 1% O_2_, 5% CO_2_, and 94% N_2_. Then, cells were cultured for 4 h at 37°C in glucose-containing medium in an atmosphere of 5% CO_2_ and 95% O_2_.

### Rat Pulmonary Microvascular Endothelial Cells Viability

Cell viability of rPMVECs infected with control or p38 MAPK shRNA before OGD/R was assayed using the CCK-8 kit (JDOJINDO, Japan) following the manufacturer’s instructions. Apoptosis was assessed using a caspase-3 spectrophotometric detection kit (Wanleibio, China) following the manufacturer’s instructions. The level of NO in cell supernatant was assessed using an NO detect kit (Beyotime, China) following the manufacturer's instructions.

### Rat Pulmonary Microvascular Endothelial Cells Permeability *In Vitro*

Cells (1 × 10^5^) were seeded into the upper chamber of polyester membranes in a 24-well Transwell (pore size 0.4 μm; diameter 6.5 mm; Costar, NY) and cultured in 0.5 ml of DMEM. Medium (1.5 ml) was added to the lower chamber, and Transwells were incubated for 2 days until the cells reached 100% confluence. The cells were subjected to the OGD/R procedure described above, and at 1 h before the end of the reoxygenation period, FITC-dextran solution was added to the upper chamber of each Transwell. Sample readings were converted to FITC-dextran concentrations based on a standard curve. The permeability coefficient (Pc) of FITC-dextran was described as Pc = [A]/t × 1/A × V/[L], where [A] denotes the concentration of FITC-dextran in the lower chamber; [L], the concentration in the upper chamber; A, the area of the membrane (cm^2^); t, time (s); and V, the volume of the lower chamber (Zhang et al., 2015).

### Immunofluorescence Staining

Cells were fixed in 4% paraformaldehyde for at least 10 min, washed three times with PBS, permeabilized with 0.5% Triton X-100, and blocked for 1 h with 5% bovine serum albumin. Then cells were incubated overnight at 4°C on a shaking table with goat polyclonal anti-ZO-1 antibody (1:100; catalog no. ab190085; Abcam) or rabbit polyclonal anti-VE-cadherin antibody (1:300; catalog no. ab33168, Abcam). PBS instead of primary antibody was used as a negative control in immunofluorescence staining. Then, the cells were exposed to an appropriate secondary antibody, donkey antigoat IgG (H + L) cross-adsorbed secondary antibody, Alexa Fluor 568 (1:1000, catalog no. A-11057, invitrogen) for ZO-1 and goat antirabbit IgG (H + L) highly cross-adsorbed secondary antibody, Alexa Fluor 568 (1:1000, catalog no. A-11036, invitrogen) for VE-cadherin, for 1 h at room temperature. Finally, cell nuclei were stained with 4′,6-diamidino-2-phenylindole (DAPI; Solarbio, China), and cells were viewed under a fluorescence microscope (Olympus BX51). Cytoskeletal changes were examined using rhodamine-conjugated phalloidin (Solarbio, China) to target F-actin.

### Real-Time qPCR

Total RNA was extracted from cells using TRIzol (catalog no. 15596-026, Invitrogen) following the manufacturer’s instructions. Total RNA (1 μg) was used as a template in a 20-μl reverse transcription reaction using the Prime Script™ RT reagent kit (Takara, Japan) following the manufacturer’s instructions. The cDNA was amplified using the following primers: p38 MAPK forward, 5'-GAA​CAA​CAT​CGT​GAA​GTG​TCA​GAA​GC-3'; p38 MAPK reverse, 5'-CCT​GTG​GAT​TAT​GTC​AGC​CGA​GTG-3'; HIF-1α forward, 5'-TCT​GGG​TTG​AAA​CTC​AAG​CAA​CTA-3'; HIF-1α reverse, 5'-CAA​CCG​GTT​TAA​GGA​CAC​ATT​CTG-3'; GAPDH forward, 5'-AGA​AGG​CTG​GGG​CTC​ATT​TG-3'; and GAPDH reverse, 5'-AGG​GGC​CAT​CCA​CAG​TCT​TC-3'.

Relative gene expression was quantified using the 2^−∆∆ct^ method relative to the expression of GAPDH.

### Western Blotting

Left lung tissues were removed and digested with 0.25% EDTA in centrifuge tubes. Digested tissues or harvested rPMVECs were homogenized in RIPA buffer (Solarbio) containing protease inhibitor cocktail (Solarbio) and phosphatase inhibitor cocktail (Cell Signaling Technology, USA) and then centrifuged at 12,000 *g* for 15 min at 4°C. The supernatant protein concentration was assayed using a Bicinchoninic acid kit (Beyotime Biotechnology), and equal amounts of proteins were separated on 10% SDS-PAGE and transferred to 0.22 μm PVDF membranes (Millipore) at 4°C. The membranes were incubated overnight at 4°C, shaking with the following primary antibodies: anti-GAPDH (1:1000, Santa Cruz), rabbit polyclonal anti-phospho-p38 MAPK (1:1000; clone D3F9, Cell Signaling Technology), rabbit polyclonal anti-p38 MAPK (1:1000; catalog no. 9212, Cell Signaling Technology), rabbit monoclonal anti-AQP1 (1:5000; catalog no. ab168387, Abcam), mouse monoclonal anti-ICAM-1 (1:2000; catalog no. ab171123, Abcam), goat polyclonal anti-ZO-1 (1:1000; catalog no. ab190085, Abcam), rabbit polyclonal anti-VE-cadherin (1:500; catalog no. ab33168, Abcam), mouse monoclonal anti-HIF-1α (5 μg/ml; catalog no. ab1, Abcam), Endothelin 1 monoclonal antibody (1:10000, catalog no. MA3-005, Invitrogen), and eNOS polyclonal antibody (1:1000, catalog no. PA1-037, Invitrogen). After being washed three times by TBST, the membranes were incubated with the appropriate secondary antibody, goat antimouse H&L IRDye@800 CW (1:10000, catalog no. ab216722), goat antirabbit H&L IRDye@800 CW (1:10000, catalog no. ab216723), or donkey antigoat H&L IRDye@800 CW (1:10000, catalog no. ab216725) for 1 h, and band density was quantified based on enhanced chemiluminescence using the Alpha Innotech system (BioRad).

### Statistical Analysis

Data were expressed as mean ± SD and analyzed using SPSS 17.0. Data were plotted using Graphpad Prism 5.0. Comparisons among multiple groups were analyzed by one-way analysis of variance, followed by the Tukey test for intergroup comparisons. Differences associated with *p <* 0.05 were considered statistically significant.

## Results

### Inhibition of p38 Mitogen-Activated Protein Kinase Mitigates Lung Ischemia Reperfusion Injury *In Vivo*

**FIGURE 1:**
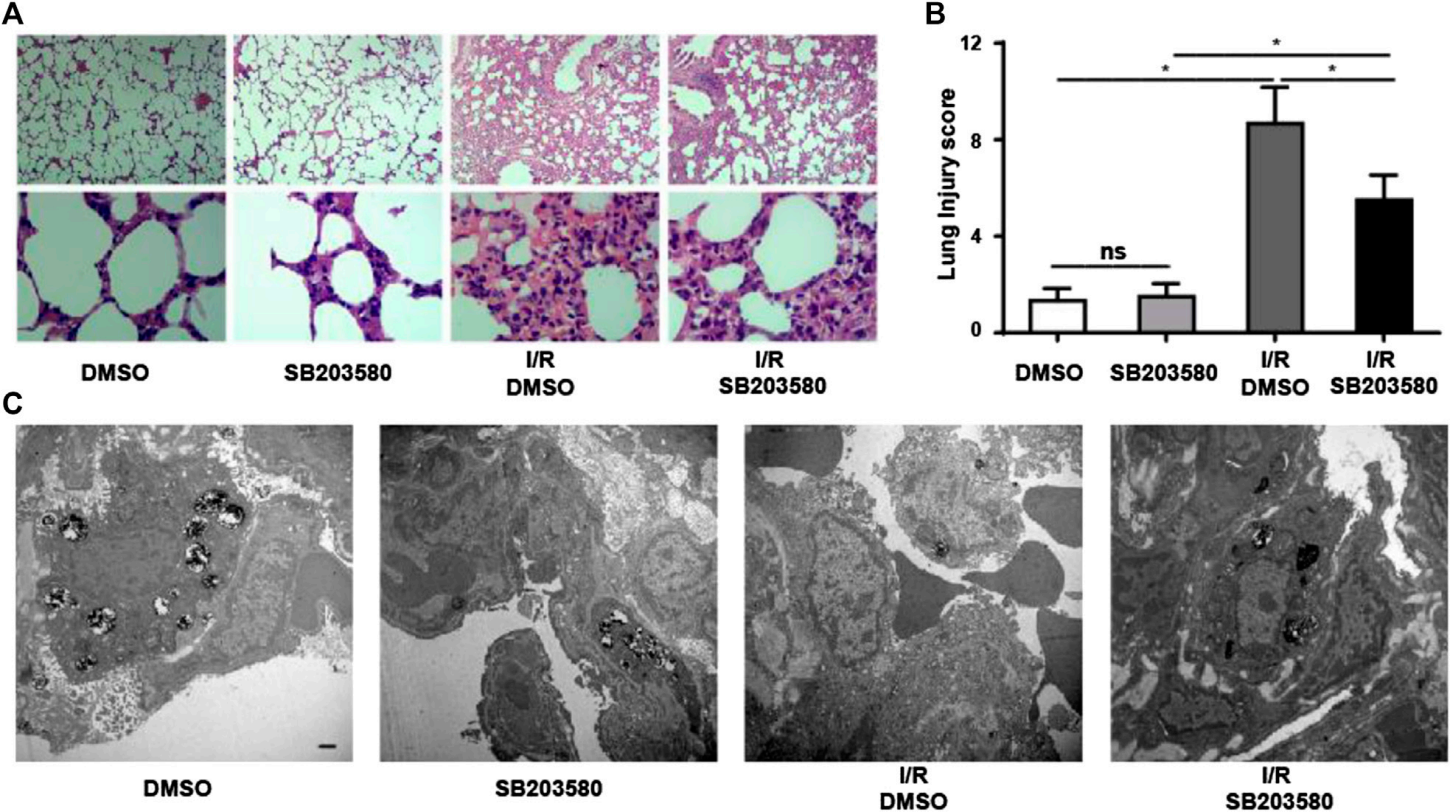
Inhibiting p38 Mitogen-activated protein kinase (MAPK) mitigates Lung ischemia reperfusion injury (LIRI) *in vivo*. **(A)** Lung sections were stained by hematoxylin and eosin and morphological changes assessed. Magnification, 40X and 400X. **(B)** Lung injury was scored based on images in panel A (*n* = 6 sections per group). ns, not significant, *p < 0.05. **(C)** Ultrastructural changes were assessed using transmission electron microscopy. Scale bar, 1 μm. Blocking P38 *MAPK in vivo* attenuates I/R-induced leakage at the pulmonary blood–air barrier

To investigate the function of p38 MAPK in an *in vivo* model of LIRI, we blocked its function using SB203580. The morphological changes in lung tissues were assessed using hematoxylin and eosin staining (Figure 1A). In the control groups, the pulmonary alveoli and interstitium were intact, and the alveolar septum showed uniform thickness without signs of alveolar wall thickening or inflammatory cell infiltration. However, the I/R group showed severe congestion and hemorrhage in the lung tissues as well as infiltration by inflammatory and red blood cells. Blocking p38 MAPK remarkably attenuated these changes (Figure 1B).

Transmission electron microscopy confirmed these results at the ultrastructural level (Figure 1C). In groups without lung injury, pulmonary endothelial cells were tightly connected with integral type II alveolar epithelial cells and an intact basement membrane, and microvilli and lamellar bodies appeared normal. In the I/R group, pulmonary endothelial cells were swollen, type II epithelial microvilli were reduced in number, and lamellar bodies were absent. These changes were partially reversed when p38 MAPK was blocked. Taken together, our results indicate that blocking p38 MAPK using SB203580 attenuates lung injury in an *in vivo* LIRI model.

**FIGURE 2:**
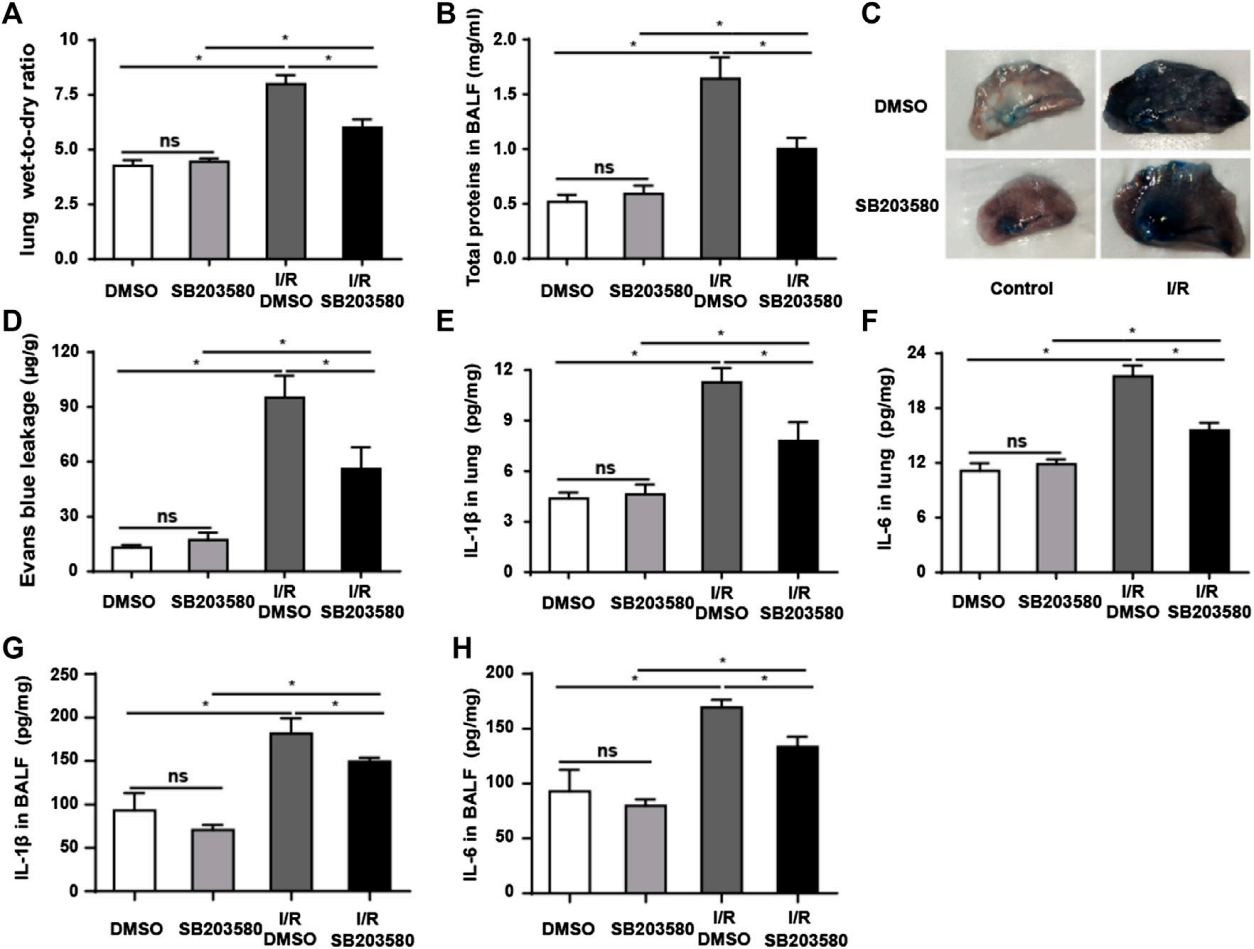
p38 MAPK inhibition attenuates I/R-induced leakage of the pulmonary blood–air barrier *in vivo*. **(A)** Wet-to-dry ratios of lung tissues as an index of lung edema. **(B)** Quantification of total protein in bronchoalveolar lavage fluid. **(C,D)** Macroscopic images of lungs and quantification of Evans blue leakage. **(E,F)** Quantification of IL-1β and IL-6 abundance in lung tissues by Enzyme-Linked Immunosorbent Assay (ELISA). **(G,H)** Quantification of IL-1β and IL-6 abundance in Bronchoalveolar Lavage Fluid (BALF) by ELISA. Data shown are mean ± SD from *n* = 7 per group. ns, not significant, *p < 0.05.

Pulmonary blood–air barrier leakage plays a crucial role in I/R-induced lung injury (de Perrot et al., 2003). We assessed lung edema by measuring pulmonary wet-to-dry ratios. I/R injury increased the ratio that blocking p38 MAPK reduced (Figure 2A). Furthermore, I/R increased the level of total proteins in BALF, and antagonizing p38 MAPK partially reversed this (Figure 2B). Consistently, Evans blue dye binding to albumin, which can be used to evaluate the permeability of pulmonary microvasculature (Zhang et al., 2020b), was significantly higher in the I/R group than in all other groups, indicating increased pulmonary microvascular leakage (Figures 2C,D). SB203580 significantly reduced the amount of Evans blue binding, indicating less leakage, which was presumably due to less injury.

The levels of IL-6 and IL-1β in lung tissues and BALF were used to evaluate the inflammatory response (Sivaraman et al., 2015; Li et al., 2020). Both were significantly higher in the I/R group in lung tissue (Figures 2E,F) and in BALF (Figures 2G,H) than in the control group, which was partially reversed by SB203580. Our findings support the idea that blocking p38 MAPK protects against the onset of pulmonary blood–air barrier leakage.

### p38 Mitogen-Activated Protein Kinase Inhibitor Attenuates I/R-Induced Changes in Endothelial Integrity Based on ZO-1 and VE-Cadherin

**FIGURE 3:**
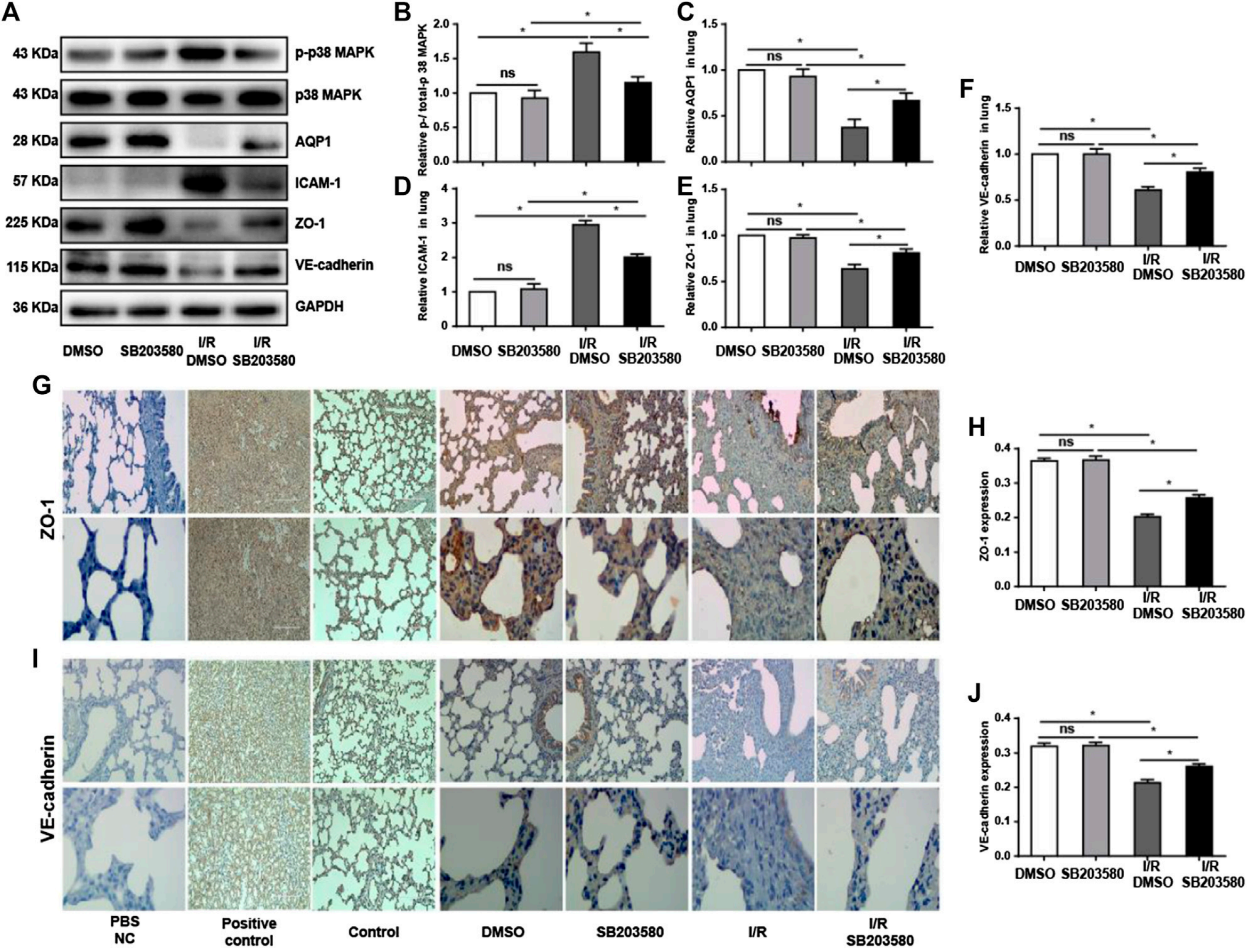
p38 MAPK inhibition attenuates I/R injury-induced damage to endothelial integrity. **(A)** Western blot of phospho-p38 MAPK, p38 MAPK, AQP1, ICAM-1, ZO-1, and VE-cadherin in lung tissues. **(B–F)** Densitometry of western blots from panel A. Levels were normalized to the control, which was defined as 1.0. **(G,I)** Immunohistochemistry of ZO-1 and VE-cadherin in lung tissues. Normal rabbit IgG in Phosphate-buffered saline (PBS) was used in the immunohistochemistry experiment instead of the primary antibody as a negative control. Rat spleen and kidney tissue instead of lung tissue were used for ZO-1 and VE-cadherin immunohistochemistry, respectively, as a positive control. Control group mice were treated without vehicle, drug, or surgery. Scale bar: 200 μm. **(H,J)** Quantification of ZO-1 and VE-cadherin immunohistochemical staining. *n* = 6 tissue sections per group. ns, not significant, *p < 0.05.

Previous studies show that the AQP1 level varies inversely with the degree of pulmonary edema (Su et al., 2004). We found that I/R injury sharply downregulated AQP1, which was reversed by SB203580 (Figures 3A–Cfig3). Disruption of endothelial cell tight junctions and adhesion junctions contributes to vascular barrier dysfunction (Yamazaki et al., 2019), and high levels of ICAM-1 can destroy endothelial junctions (Clark et al., 2007). Thus, we examined the expression of ZO-1, a marker of endothelial tight junctions; VE-cadherin, a marker of endothelial adhesion junction (Li et al., 2020); and ICAM-1 to assess the extent of endothelial barrier destruction. I/R significantly upregulated ICAM-1 and downregulated ZO-1 and VE-cadherin, which SB203580 partially reversed (Figures 3A,D–F).

The results with western blotting were confirmed using immunofluorescence (Figures 3G,I), which also showed that ZO-1 and VE-cadherin protein levels were markedly decreased after I/R injury compared with control groups although pretreatment with SB203580 increased their expression. These findings suggest that inhibition of p38 MAPK can significantly alleviate I/R-induced destruction of the endothelial barrier.

### Inhibition of p38 Mitogen-Activated Protein Kinase protects rPMVECs from Oxygen/Glucose Deprivation and Reoxygenation Injury

**FIGURE 4:**
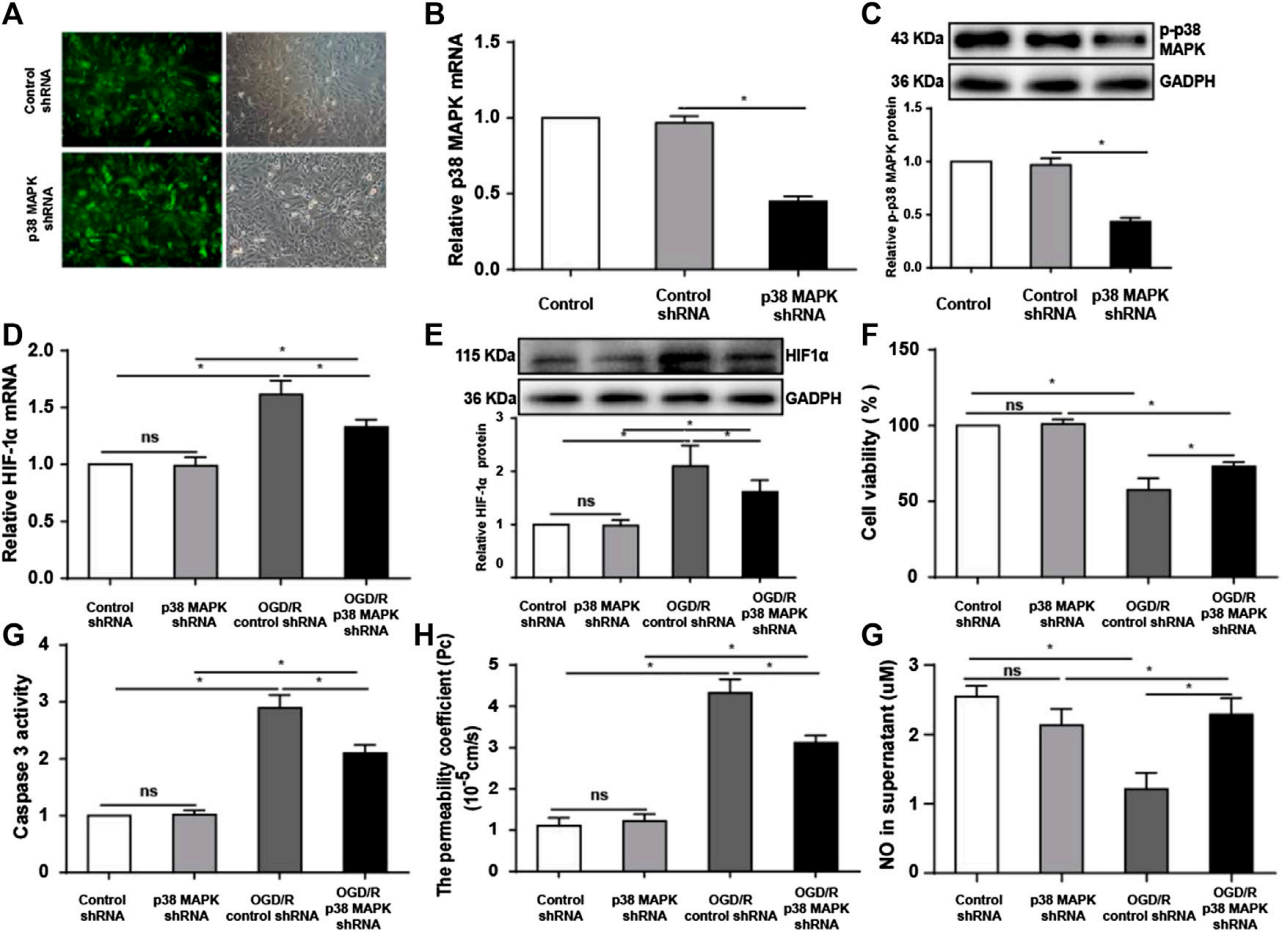
Inhibiting p38 MAPK protects rPMVECs from OGD/R injury. **(A)** Fluorescent and bright field micrographs of rPMVECs transfected with lentivirus-expressing control or p38 MAPK-targeting shRNA. Levels of **(B)** p38 MAPK mRNA, **(C)** p-p38 MAPK protein, **(D)** HIF-1α mRNA, and **(E)** HIF-1α protein were measured. **(F)** Viability of rPMVECs was measured using the CCK-8 assay. **(G)** Activity of caspase 3 in rPMVECs was measured as an index of apoptosis. **(H)** The permeability coefficient in rPMVECs was measured as an index of the pulmonary blood–air barrier based on flux of the fluorescent marker FITC-dextran. **(G)** The level of NO in cultured rPMVEC supernatant in different groups was detected. ns: no statistical significance, *p < 0.05, each experiment was performed at least three times independently. Blocking p38 MAPK mitigates OGD/R-induced endothelial dysfunction.

To gain mechanistic insights into our results with the *in vivo* model, we moved into experiments in rPMVECs. First, we confirmed that we could knock down p38 MAPK in this cell line. Cells were transfected with a lentiviral vector expressing shRNA targeting p38 MAPK. Green fluorescence indicated a transfection efficiency greater than 80% (Figures 4A). The shRNA targeting p38 MAPK led to significantly lower levels of p38 MAPK mRNA and phospho-p38 MAPK than the control shRNA (Figures 4B,C). Next, we confirmed that we could induce OGD/R in the cells as a disease model as described (Shen et al., 2004). HIF-1α is a crucial transcriptional factor induced by hypoxia conditions (Ryou et al., 2015). As expected, OGD/R upregulated HIF-1α. This upregulation was partially reversed by p38 MAPK knockdown (Figures 4D,E).

In addition, OGD/R reduced cell viability by 50% (Figure 4F) and upregulated pro-apoptotic caspase-3 activity (West et al., 2006) (Figure 4G), both of which were reversed by p38 MAPK knockdown. OGD/R markedly increased FITC-dextran flux (Figure 4H), indicating hyperpermeability of the pulmonary blood–air barrier (Hoffmann et al., 2011), which p38 MAPK knockdown significantly decreased. Regarding endothelial dysfunction, OGD/R decreased the NO level in the cell supernatant, and p38 MAPK knockdown attenuated the decrease (Figure 4G). Together, these results indicate that p38 MAPK knockdown can protect against OGD/R injury.

**FIGURE 5:**
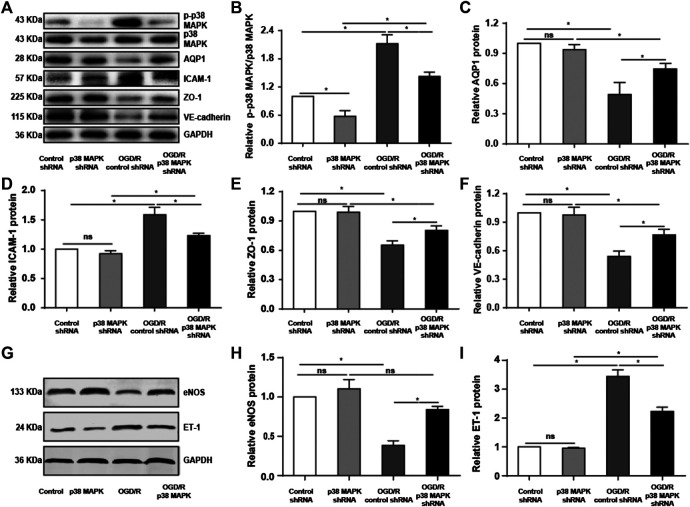
Knocking down p38 MAPK protects against endothelial destruction induced by OGD/R. **(A)** Western blot of phospho-p38 MAPK, p38 MAPK, AQP1, ICAM-1, ZO-1, and VE-cadherin in rPMVECs. **(B–F)** Quantification of proteins from western blots in panel A. **(G)** Western blot of eNOS and ET-1 in rPMVECs. **(H,I)** Quantification of proteins from western blots in panel G. Each experiment was performed independently at least three times. ns, not significant, *p < 0.05.

To confirm our *in vivo* findings, we measured levels of phospho-p38 MAPK, AQP1, ICAM-1, VE-cadherin, and ZO-1 expression in rPMVECs. OGD/R upregulated phospho-p38 MAPK and ICAM-1, which p38 MAPK knockdown reversed (Figures 5A,B,D). Conversely, OGD/R downregulated AQP1, ZO-1, and VE-cadherin, which p38 MAPK knockdown reversed (Figures 5A,C,E,F). Also, we found that OGD/R promoted endothelial inflammation and dysfunction indicated by the decrease of eNOS and increase of ET-1, which p38 MAPK knockdown reversed (Figures 5G–I). These results support our *in vivo* findings that OGR/D induces endothelial inflammatory damage and compromises the permeability barrier, which inhibition of p38 MAPK can mitigate.

### p38 Mitogen-Activated Protein Kinase inhibition mitigates Oxygen/Glucose Deprivation and Reoxygenation-Induced Changes In Expression of ZO-1 and VE-Cadherin and in F-Actin Distribution

**FIGURE 6:**
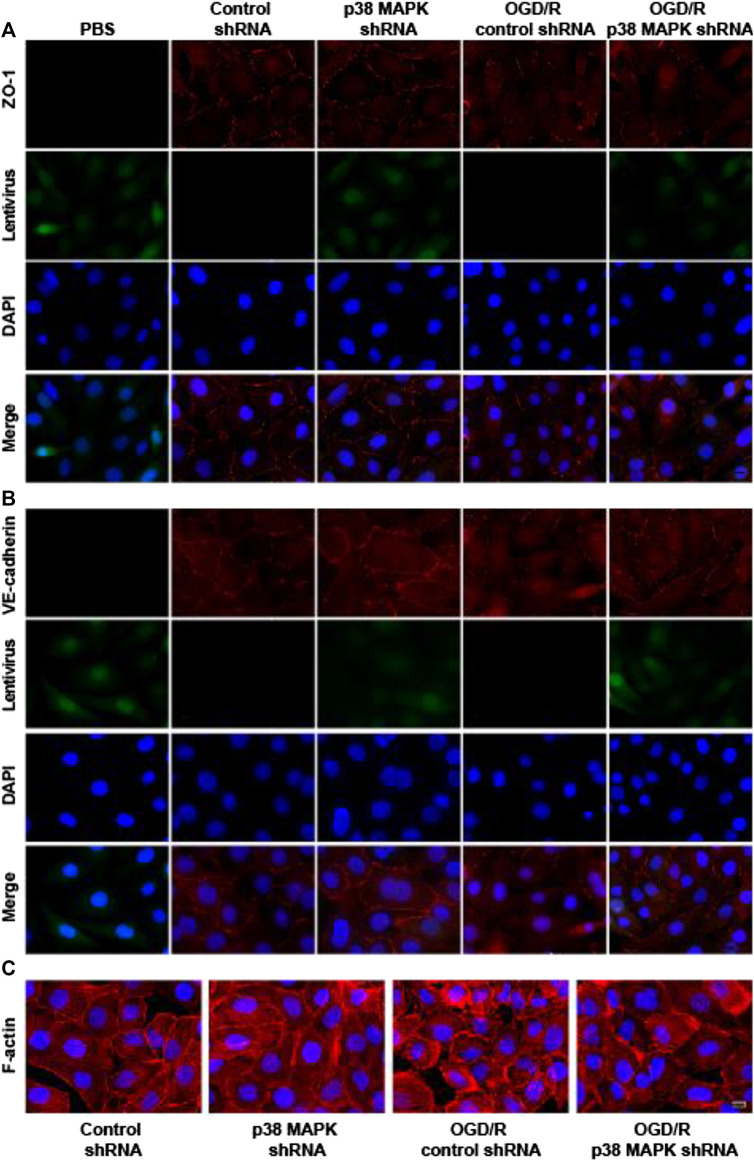
p38 MAPK knockdown mitigates OGD/R-induced changes in expression of ZO-1 and VE-cadherin and in F-actin distribution. **(A–C)** Immunofluorescence micrographs showing ZO-1, VE-cadherin, and F-actin in rPMVECs. PBS was used instead of the primary antibody as a negative control in the immunofluorescence experiment. Green indicates lentivirus. Nuclei were stained with DAPI (blue). Scale bar, 10 μm.

To further confirm the role of p38 in mediating endothelial destruction induced by OGD/R, we performed immunofluorescence staining, which showed that OGD/R downregulated ZO-1 and VE-cadherin, while p38 MAPK knockdown reversed this (Figures 6A,B). In control rPMVECs, F-actin localized mainly on the cell membrane such that cell borders were obvious. OGD/R led to accumulation of F-actin stress fibers and blurred cell boundaries, which p38 MAPK knockdown mitigated. These results suggest that inhibition of p38 MAPK preserves expression of proteins that maintain the endothelial barrier.

## Discussion

Inhibition of p38 MAPK has been shown to therapeutically treat acute lung injury induced by intestinal I/R in rats (Xiong et al., 2016) or by swine influenza virus or lipopolysaccharide in mice (Gao et al., 2013; Wei et al., 2014). Therefore, in the present study, we explored the potential therapeutic effect of inhibiting p38 MAPK against LIRI in rats and OGD/R injury in rPMVEC cultures. We found that the rats subjected to LIRI exhibited severe pathological changes in lung morphology and ultrastructure, and rPMVECs subjected to OGD/R strongly upregulated HIF-1α (Ryou et al., 2015), indicating that we established both disease models. In both models, inhibition or knockdown of p38 MAPK mitigated injury, suggesting that p38 MAPK contributes to tissue damage during LIRI and may be a good therapeutic target.

The blood–air barrier contributes to pulmonary gas exchange (Bärnthaler et al., 2017), which is compromised during LIRI when the microvascular endothelial layer is damaged (Zhang et al., 2015). Subsequent inflammatory cell infiltration and edema are important features of acute lung injury (Herold et al., 2013) as well as inflammation driven by IL-1β and IL-6 (Bosmann and Ward, 2013). Consistently, I/R *in vivo* and OGD/R *in vitro* both increased the high permeability of the endothelial barrier, which may be driven by the increased level of IL-1β and IL-6 and contribute to inflammatory cell infiltration and edema. Inhibition of p38 MAPK attenuated the increase of IL-1β and IL-6 in the lung and BALF, providing a potential target for inflammation and blood–air barrier damage during LIRI.

Edema resulting from lung injury is inversely related to levels of AQP1, which can reduce lung edema (Han et al., 2015; Xu et al., 2016; Li et al., 2018). Consistently, we found that the level of AQP1 expression decreased due to injury *in vivo* and *in vitro* but was partially restored when p38 MAPK was inhibited. Conversely, ICAM-1 contributes to injury by altering cell junctions or the cytoskeleton to increase microvascular endothelial cell permeability (Clark et al., 2007). In rats exposed to lung I/R, knockdown of p38 MAPK downregulated ICAM-1 and mitigated the resulting injury (Lv et al., 2012). In our study, we detected increased levels of ICAM-1 at sites of structural destruction, and we found that p38 MAPK inhibition or knockdown attenuated I/R-induced upregulation of ICAM-1 *in vivo* and *in vitro*, which was associated with less severe damage to cellular morphology and tight junctions. Collectively, our results illustrate that knockdown of p38 MAPK can affect positive and negative regulators of endothelial architecture.

Likewise, we found that p38 MAPK can regulate specific mediators of tight junctions that maintain the endothelial cell barrier. ZO-1 forms part of the endothelial tight junctions and helps maintain the integrity of the endothelial barrier (Tornavaca et al., 2015). VE-cadherin is an adhesion protein located specifically at the tight junctions of endothelial cells (Wu et al., 2016), and F-actin is the main cytoskeletal contraction protein, which is important for maintaining the morphology and normal function of cells (Deng et al., 2018). I/R induced the degradation of tight junctions and the accumulation of F-actin stress fibers, which results in the loss of endothelial barrier function (Deng et al., 2018). We found that ZO-1 and VE-cadherin were dramatically downregulated by LIRI or OGD/R and that p38 MAPK inhibition reversed these effects. Our results are consistent with previous work linking p38 MAPK to ZO-1. Inhibition of p38 MAPK has been shown to increase the expression of ZO-1, thereby alleviating dysfunction of the human endothelial barrier induced by high mobility group tbox1 protein (Luan et al., 2018). *Clostridium tyrobutyricum* inhibits the p38 MAPK signaling pathway to upregulate ZO-1 and thereby protect against dysfunction of the intestinal barrier induced by lipopolysaccharide (Xiao et al., 2018). Our results and previous studies suggest that inhibition of p38 MAPK can protect tight junctions and the cytoskeleton to maintain the integrity of the endothelial barrier in the face of I/R injury.

Our study with *in vivo* and *in vitro* injury models suggests that inhibiting p38 MAPK mitigates LIRI and I/R-induced inflammatory responses at least in part by restoring the integrity of the pulmonary blood–air barrier. Such inhibition may work by upregulating AQP1 and downregulating ICAM-1. Our findings support antagonizing p38 MAPK as a new therapeutic approach to limit LIRI and, ultimately, acute lung injury.

## Data Availability Statement

The raw data supporting the conclusions of this article will be made available by the authors, without undue reservation, to any qualified researcher.

## Ethics Statement

The animal study was reviewed and approved by Guangxi Medical University Institutional Animal Care and Use Committee.

## Author Contributions

TW curated data and wrote the original draft of the manuscript. CL handled the animal model, IHC, WB, and QPCR experiments and wrote part of the manuscript. L-hP analyzed data. ZL collected microscopy images. C-lL performed a literature search, and J-yL performed statistical analysis. YH collected data, and J-yX handled the animal model. SW and YQ did QPCR and WB. ZL and FL wrote and edited the manuscript. FL conceptualized and managed the project.

## Funding

This study was supported by the National Natural Science Foundation of China (81560018, 8196022, and 81900234), the 2017 Young Anesthesiologist Research Fund of the Anesthesiology Branch of the Chinese Medical Doctor Association, the Guangxi Thousands of Young and Middle-aged Backbone Teacher Training Program and Guangxi Medical High-level Talents Program (G201903011), the Postdoctoral Innovative Talent Support Program (BX20180393), and the China Postdoctoral Science Foundation (2018M640859).

## Conflict of Interest

The authors declare that the research was conducted in the absence of any commercial or financial relationships that could be construed as a potential conflict of interest.
